# Synergy and Attenuation of Work-Related Factors in Musculoskeletal Disorders: The Combined Risk Based on Data from the Korean Working Conditions Survey

**DOI:** 10.3390/medicina61111969

**Published:** 2025-11-02

**Authors:** Eun-Soo Lee, Myong-Hwan Kim, Dongmug Kang, Youn-Hyang Lee, Yoon-Ji Kim, Se-Yeong Kim, Youngki Kim

**Affiliations:** 1 Department of Occupational and Environmental Medicine, Pusan National University Yangsan Hospital, Yangsan 50612, Republic of Korea; es3003@naver.com (E.-S.L.); danielk@pusan.ac.kr (M.-H.K.); kangdm@pusan.ac.kr (D.K.); 30white@pusan.ac.kr (S.-Y.K.); 2 Department of Preventive, and Occupational & Environmental Medicine, School of Medicine, Pusan National University, Yangsan 50612, Republic of Korea; harry-potter-79@hanmail.net; 3 Research Institute for Convergence of Biomedical Science and Technology, Pusan National University Yangsan Hospital, Yangsan 50612, Republic of Korea; 4 Department of Nursing, Choonhae Health Sciences University, Ulsan 44965, Republic of Korea; llh113@nate.com

**Keywords:** Job Exposure Matrix, job strain, interaction, statistical, sex factors, ergonomics, surveys and questionnaires

## Abstract

*Background and objectives:* Musculoskeletal disorders (MSDs) account for more than 60% of compensated occupational diseases in Korea. Despite this burden, benchmarks of standardized ergonomic exposure and evidence on the combined effects of risk factors remain limited. This study aimed to construct a body part-specific ergonomic job exposure matrix (JEM) and evaluate the independent and interactive effects of ergonomic, demographic, and work-related factors. *Materials and Methods:* We analyzed the data of 210,500 workers from the 2nd–6th Korean Working Conditions Survey (2009–2020). A JEM for arms/neck, back, and legs was developed and validated (κ ≥ 0.79). Logistic regression models estimated adjusted odds ratios (aORs), and additive interactions were assessed using relative excess risk due to interaction (RERI), attributable proportion (AP), and the synergy index (SI). *Results:* High ergonomic exposure was strongly associated with MSDs across all body regions (aORs 2.3–2.5). Age > 45 years, long working hours (>52 h), and high job strain also increased risks (aORs 1.4–2.3). On the additive scale, ergonomic risk combined with older age showed consistent synergy (RERI up to 1.5; SI >1.5), whereas combinations with long working hours or job strain showed attenuation (RERI < 0; SI < 1). Women reported higher crude prevalence but lower adjusted odds (aOR ≈ 0.9). *Conclusions:* This nationally representative study demonstrates that ergonomic risk, age, long working hours, and job strain are major determinants of MSDs. The validated Korean JEM provides a standardized tool for surveillance and compensation. However, the cross-sectional design limits causal inference. Future longitudinal research with objective exposure measures is needed to strengthen causal inference and guide tailored prevention.

## 1. Introduction

Musculoskeletal disorders (MSDs) are among the most common occupational health problems worldwide, and impose substantial medical, social, and economic burdens [1]. In Korea, MSDs represent the most frequently compensated occupational disease, accounting for more than 60% of all recognized cases [2]. Identifying high-risk groups and occupations is therefore crucial for developing prevention strategies and shaping occupational health policies.

Although MSDs develop through multifactorial mechanisms, several domains of risk have been consistently identified. *First, physical/ergonomic load*—including repetitive motion, forceful exertion, awkward postures, and manual material handling—is a principal driver of MSDs across body regions [1]. *Second, individual demographic factors* such as gender and age modify MSD susceptibility; women often report a higher prevalence than men, even in comparable jobs, reflecting both biological and organizational differences (e.g., task allocation, lower job control) [3]. *Third, psychosocial stressors*—notably high job demands, low support, and perceived job insecurity—contribute to symptom onset and persistence [4]. *Fourth, organizational factors* such as long working hours further exacerbate the risk. In Korea, despite the statutory 52 h limit, long workweeks remain common and are associated with greater risk of musculoskeletal symptoms [5], a pattern echoed in international reviews and meta-analyses [6,7].

*Compared to other hazards, exposure assessment for MSDs has not been accurately developed in Korea*. Job exposure matrices (JEMs) are well developed for chemical [8], physical [9], and psychosocial risks [10], but MSD-specific JEMs remain limited. The French Cohorte des consultants des Centres d’examens de santé (CONSTANCES) JEM integrates ergonomic information across hundreds of job codes, yet does not distinguish exposures by anatomical site [11]. Similarly, the Helsinki Health Study JEM covers a narrower set of exposures or occupations [12]. A comprehensive, body part-specific ergonomic JEM applicable across all occupations is still lacking.

*Beyond the primary effects, rigorous examination of interactions (effect modification) is crucial.* Epidemiologic guidance recommends assessing both additive and multiplicative scales of interaction to clarify causality and public-health impact [13]. Ergonomic load may combine with psychosocial strain or long working hours to produce risks greater than expected from either factor alone. However, nationally representative evidence on whether these combined effects are additive, multiplicative, or supra-multiplicative remains limited.

Taken together, substantial gaps persist in understanding the main effects of ergonomic, demographic, psychosocial, and organizational factors *and their interactions*, as well as in the availability of body part-specific ergonomic exposure tools. The Korean workforce—characterized by a high MSD burden [2], persistent long working hours [5,6,7], and the availability of rich national survey resource data [14]—offers a unique setting to address these gaps.

Therefore, this study aimed: (1) To estimate the body region wise prevalence of MSD symptoms (arm/neck, back, and legs) in the Korean workforce, stratified by sex; (2) To construct a nationally applicable, body part-specific ergonomic JEM; and (3) To examine both independent effects and interactions among sex, psychosocial stress, working hours, and ergonomic exposures.

## 2. Materials and Methods

### 2.1. Data Sources and Study Population

The Korean Working Conditions Survey (KWCS) has been carried out six times since 2006. This survey was developed based on the European Working Conditions Survey (EWCS) and is conducted triennially to examine the overall working environment, including the type of work, employment, occupation, industry, exposure to risk factors, and employment stability, among workers aged 15 years or older in Korea. The sample for the Working Environment Survey is selected from census data, stratified by region, and consists of 50,000 households nationwide. Investigators visit the selected households to collect data through one-on-one interviews, and participation is voluntary. The first and second KWCS surveyed only 10,000 people, but 50,000 people have been surveyed since the third KWCS. In this study, data from the 2nd to 6th KWCSs were used. Data from the 1st KWCS, which used a different occupational classification system, has been excluded. A total of 210,500 people were finally selected as subjects, excluding people whose occupations were difficult to classify. The subjects included 9991 participants from the 2nd KWCS, 49,957 participants from the 3rd KWCS, 49,905 participants from the 4th KWCS, 50,110 participants from the 5th KWCS, and 50,537 participants from the 6th KWCS.

### 2.2. Job Classification

In this study, the job classification was based on the Korean Standard Classification of Occupations (KSCO) developed by Statistics Korea. Initially, the KWCS data categorized occupations using either the unit group or the detailed occupational categories of the 6th KSCO. Subsequently, for this study, these classifications were updated to align with the unit group categories of the 7th KSCO, the updated version of which was published in 2017.

### 2.3. Ergonomic Risks of Arm/Neck, Back, and Legs

To assess the ergonomic risks related to work-related MSDs, questionnaire items about local vibrations among the physical risk factors and ergonomic factors were used. To identify MSD risk factors related to the arms and neck, the questions covered the following: ① Vibrations from hand tools or machines, ② Postures that cause fatigue or pain, and ③ Repetitive hand or arm movements. To identify the MSD risk factors associated with the lower back, the questions covered the following: ① Lifting, pushing, or carrying heavy objects, ② Postures that cause fatigue or pain, and ③ Lifting or moving people. The MSD risk factors associated with the legs were examined using questions related to ① Lifting, pushing, or moving heavy objects and ② prolonged standing posture. The exposure intensity was measured on a 7-point scale. The duration of exposure to risk factors was classified as “high”, “moderate”, or “low”. When the exposure encompassed the entire working hour period or most or 3/4th of the working hours, the responses were classified as “high exposure”, and when the duration of exposure was 1/2 or 1/4th of the total working hours, the responses were classified as “moderate exposure”. When the subjects responded, “rarely exposed/never exposed”, these cases were categorized as “low exposure”.

### 2.4. Development and Validation of the Job Exposure Matrix (JEM) for Ergonomic Risks to Body Parts (Arms and Neck, Back, Legs)

To evaluate the body part-specific ergonomic risk by occupation in the Korean workforce, a JEM was constructed using questionnaire items from the KWCS. The items were selected to reflect exposure to musculoskeletal risk factors, with three items pertaining to the arms and neck (hand–arm vibration, awkward postures, and repetitive movements), three for the lower back (heavy lifting, awkward postures, and moving people), and two for the legs (heavy lifting and prolonged standing).

The individual-level exposure intensity was measured on a 7-point scale. If when the duration of exposure to risk factors is the total working hours or most or 3/4 of the working hours, the responses were classified as ‘high exposure, and when the duration of exposure was half or 1/4 of the working hours, the responses were classified as ‘moderate exposure.’ When the subjects answered ‘rarely exposed/never exposed’, these cases were categorized as ‘low exposure.’ Individual-level exposure intensity was determined by combining the responses for each body part. If at least one item was rated as “high” (i.e., exposure for most or all of the working hours), the body part was classified as high exposure. If at least two items were rated as “moderate,” the region was classified as moderate exposure. All other combinations were considered low exposure. These individual responses were then aggregated by job category. For each occupation and body part, if 60% or more of workers were classified as having high exposure, the occupation was assigned a high exposure level for that region. Similarly, if 60% or more were classified as low exposure, the occupation was assigned a low exposure level; all remaining cases were assigned moderate exposure.

This occupation-based classification of exposure levels for each anatomical region constituted the JEM. The JEM was used as an analytic tool to examine occupational patterns of exposure and their association with the prevalence of musculoskeletal symptoms.

To assess the validity of this JEM, two ergonomics experts independently evaluated the exposure intensity for each body part by job category. The experts were provided with the relevant KWCS items, KSCO job titles, and job descriptions. Discrepancies between the two assessments were resolved through consensus discussions. Agreement between the expert consensus ratings and the JEM-based estimates was assessed using Cohen’s kappa coefficient. For the arms and neck, as well as the lower back, the level of agreement was high (κ ≥ 0.80), supporting the validity of the JEM classification. For the legs, the level of agreement was substantial (κ = 0.79) [15].

### 2.5. Job Stress and Working Hours/Week

We measured psychosocial job stress with *a demand–control* construct harmonized to the EWCS. Demand was assessed with two items evaluating the following: (i) whether the job involves *working at very high speed*, and (ii) whether respondents *have enough time to get the job done* (five-point frequency scales). We classified the job as *high demand* if the respondent reported working at a very high speed for *three-quarters of the time or more, or* reported *insufficient time* (*rarely/never* enough time). Control was assessed based on two items, namely, *being able to take a break when desired and being able to apply one’s own ideas in the work.* The job was classified as *high control* when *both* items were answered *always/most of the time* (otherwise *low control*). From these, we defined *job strain* (Karasek) as *high demand combined with low control* (binary variable *DCM_high* = 1; all other combinations = 0). These derived indicators were created prior to modeling and used consistently in descriptive and regression analyses.

Weekly working hours were categorized as >52 h/week versus ≤52 h/week. All analysis variables were harmonized to binary coding with the lower-risk category as the reference. In addition to the overall cohort, sex-stratified analyses were conducted.

### 2.6. Musculoskeletal Symptoms

Musculoskeletal symptoms were examined based on responses to questions about the presence or absence of arm (upper extremity) and neck, low back, and leg (lower extremity) pain.

### 2.7. Statistical Analysis

#### 2.7.1. Descriptive Analyses

We summarized participant characteristics by sex. Categorical variables were compared using the χ^2^ test; continuous variables, using the *t*-test. Two-sided *α* = 0.05 defined statistical significance. Analyses were performed with SAS 9.4 (SAS Institute Inc., Cary, NC, USA).

#### 2.7.2. Multivariable Logistic Regression (Primary Analyses)

For each outcome (arm/neck pain, back pain, leg pain), we fitted multivariable logistic regression models including the region-specific ergonomic risk and the following covariates chosen a priori: sex, age (>45 vs. ≤45 years), long working hours (>52 vs. ≤52 h/week), and job strain (high vs. low). Adjusted associations were reported as odds ratios (aORs) with 95% confidence intervals (CIs).

*Model building and selection*: For each body region, we estimated: (i) A simple (bivariable) model with the exposure of interest only; (ii) A prespecified full model containing all the main effects plus selected two-way product terms (ergonomic risk × age, ergonomic risk × long hours, ergonomic risk × job strain, age × long hours, age × job strain, and long hours × job strain), and—when analyzing the combined sample—sex × (ergonomic risk/age/long hours/job strain); and (iii) A backward-elimination model starting from the full model, retaining all main effects and removing non-retained interactions (*p* ≥ 0.05) as a sensitivity analysis. To ensure comparability across outcomes and sexes and to avoid variable-selection bias, the full model was designated as the primary specification; the simple and backward models were used to assess robustness.

*Multicollinearity diagnostics*: We computed variance inflation factors (VIFs) for all main-effect predictors. For models with interaction terms, the components of cross-products were mean-centered, and the VIFs were compared with conventional thresholds (≈5–10) to evaluate the concern for multicollinearity. Results did not indicate problematic collinearity.

### 2.8. Interactions Between Risk Factors

#### 2.8.1. Multiplicative Interaction

To assess whether effects differed by sex, age, long working hours, or job strain, we added the product terms described above to the logistic models and tested them using Wald χ^2^ statistics (two-sided α = 0.05). Interpretation followed standard guidance on presenting effect modification on the multiplicative scale [13].

#### 2.8.2. Additive Interaction

Because interaction is scale-dependent, we additionally quantified interaction on the additive scale using the relative excess risk due to interaction (RERI), the attributable proportion due to interaction (AP), and the synergy index (SI) [13,16]. Risk ratios (RRs) for the four joint exposure strata were estimated via modified Poisson regression with a log link and robust (scale-corrected) variance. We then computed, within text:

RERI = RR11 − RR10 − RR01 + 1;

AP = RERI/RR11;

SI = (RR11 − 1)/[(RR10 − 1) + (RR01 − 1)].

Delta-method standard errors and 95% CIs were derived from model covariance matrices. We interpreted additive interaction measures using conventional thresholds:

RERI, AP: 0 as the null value; CIs excluding 0 indicate significant interaction.

SI: 1 as the null value; CIs excluding 1 indicate a significant interaction.

Thus, RERI > 0, AP > 0, or SI > 1 with CIs excluding the null indicate synergistic interaction, whereas RERI < 0, AP < 0, or SI < 1 with CIs excluding the null indicate antagonistic (sub-additive) interaction. When individual exposures were associated with increased risk (odds ratios [ORs] or risk ratios [RRs] >1) but additive measures indicated antagonism, we interpreted this not as a protective joint effect but as attenuation or sub-additivity of the combined effect, suggesting that the joint impact was weaker than expected under additivity.

Although all three indices evaluate additive interaction, each emphasizes a different perspective: RERI quantifies the *absolute excess risk* attributable to interaction, AP expresses the *proportion of the total risk* among those with both exposures due to their interaction, and SI measures the *relative strength* of interaction compared with independent effects. Because these indices provide complementary information, it is recommended that all three measures be presented when reporting additive interaction [13,16].

## 3. Results

### 3.1. General Characteristics of the Subjects and Distribution of the Study Variables

**Figure 1:**
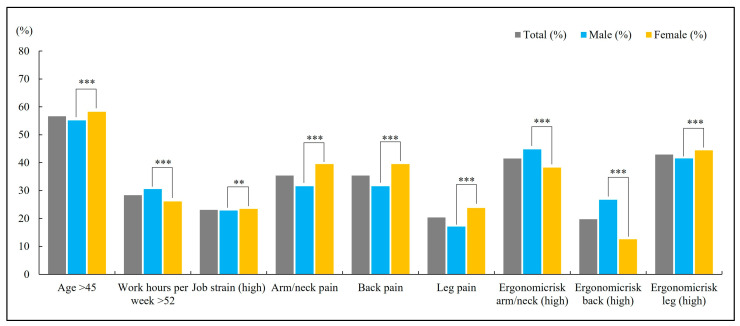
Distribution of study variables by sex (χ^2^ test). Bars represent percentages within each subgroup (Total, Male, Female). Total n = 210,500; Male n = 106,467 (50.6%); Female n = 104,033 (49.4%). Stars indicate the significance of the male–female comparison (Chi-square test): ** *p* < 0.01, *** *p* < 0.0001.

The study included a total of 210,500 participants: 106,467 men (50.6%) and 104,033 women (49.4%). Figure 1 illustrates the distribution of study variables. Overall, 56.6% of participants were older than 45 years, and 28.3% worked more than 52 h/week. The crude prevalence of musculoskeletal symptoms was 35.4%, 20.3%, and 24.7% for arm/neck, back, and leg pain, respectively. Most comparisons by sex were statistically significant (χ^2^; *p* < 0.001). Women were slightly older (≥45 years: 58.2% vs. 55.1%) but less often worked >52 h/week (26.1% vs. 30.5%). They reported higher crude prevalence of pain at all three sites—arm/neck (39.5% vs. 31.5%), back (23.7% vs. 17.0%), and legs (28.8% vs. 20.6%). Ergonomic risk patterns varied by body region: men were more often in the high-risk categories for arm/neck (44.7% vs. 38.2%) and back (26.7% vs. 12.5%), whereas women were more often in the high-risk category for legs (44.4% vs. 41.5%). Women also had a slightly higher frequency of high job strain (23.4% vs. 22.8%).

### 3.2. Occupations with a High Prevalence of Musculoskeletal Pain by Exposure Level

We identified 27 occupations with the maximum ergonomic exposure score (i.e., total score = 6, comprising 2 points each for all three body parts). Table 1 presents the occupations by order of prevalence of musculoskeletal pain in all three body parts. The highest prevalence of multi-region MSD symptoms was observed among crop growers (32.9%), followed by agriculture, forestry, and fishery-related elementary workers (31.7%) and fishery-related workers (31.1%). A high proportion of workers in construction-related occupations, such as construction-related technical workers (25.8%) and construction and mining laborers (18.6%), also exhibited musculoskeletal symptoms across all three body parts. Other occupational groups with relatively high symptom prevalence included cleaners and sanitation workers (22.6%), livestock and stockbreeding related workers (19.9%), and ship workers (18.2%).

### 3.3. Multivariable Associations with Musculoskeletal Pain (Arm/Neck, Back, Legs) and Risk Factors

Table 2 presents adjusted odds ratios (aORs, 95% CIs) and interaction terms for arm/neck, back, and leg pain. In the overall sample, a higher ergonomic risk for the relevant body region showed a positive association with symptoms: compared with low risk, high risk was associated with increased odds of arm/neck pain (aOR 2.49, 95% CI 2.38–2.60), back pain (2.42, 2.30–2.55), and leg pain (2.51, 2.36–2.66). Age > 45 years was also positively associated with symptoms (arm/neck: 1.57, 1.51–1.63; back: 1.54, 1.47–1.62; leg: 1.79, 1.71–1.86). Long working hours (>52 h/week) were associated with increased odds across outcomes (arm/neck: 1.71; back: 1.72; leg: 1.54), as was high job strain (arm/neck: 1.55, 1.48–1.63; back: 1.54, 1.45–1.63; leg: 1.38, 1.31–1.46).

Sex-stratified models showed broadly consistent patterns for ergonomic risk and age. Among men, high ergonomic risk was associated with increased odds of arm/neck (1.96, 1.84–2.10), back (2.04, 1.89–2.20), and leg pain (2.00, 1.74–2.30); among women, the corresponding associations were likewise positive (arm/neck: 2.34, 2.23–2.46; back: 2.42, 2.26–2.58; leg: 2.33, 2.19–2.48). Age > 45 years remained positively associated with all symptoms in both men (2.03, 2.08, 2.67) and women (2.12, 2.11, 2.25). Long working hours showed increased odds in both sexes, with a larger magnitude in women (2.18–2.27) than in men (1.39–1.51). High job strain was positively associated with symptoms in men (1.29–1.36) and women (1.48–1.80). Notably, after adjustment, women had lower odds of symptoms than men (overall models: aOR 0.91 for arm/neck and back; 0.94 for leg), despite higher crude prevalence in women.

*Interaction terms showed coherent patterns*. *Ergonomic risk × age displayed a positive association overall (1.30–1.37) and within sex strata (men: up to 1.69 for leg; women: 1.45–1.54), indicating stronger effects of ergonomic risk at older ages*. By contrast, ergonomic risk × long working hours and ergonomic risk × high job strain showed inverse associations overall (≈0.75–0.84) and within sex strata (≈0.63–0.78 in men; ≈0.69–0.74 in women). Age × long working hours and age × high job strain also showed inverse associations (overall ≈ 0.89–0.91; men ≈ 0.82–0.92; women ≈ 0.68–0.83). *The job strain × long working hours term was near null overall for arm/neck and back (≈1.00) and slightly positive for leg (1.07, 1.00–1.14); it was generally not significant in men and inverse in women for arm/neck and back (0.94 and 0.90, respectively; leg near null)*. Additional sex interactions indicated a positive association for sex × age (1.37–1.55) and sex × long working hours (1.05–1.34) and an inverse association for sex × job strain (0.85–0.93).

### 3.4. Interactions Between Risk Factors

**Figure 2:**
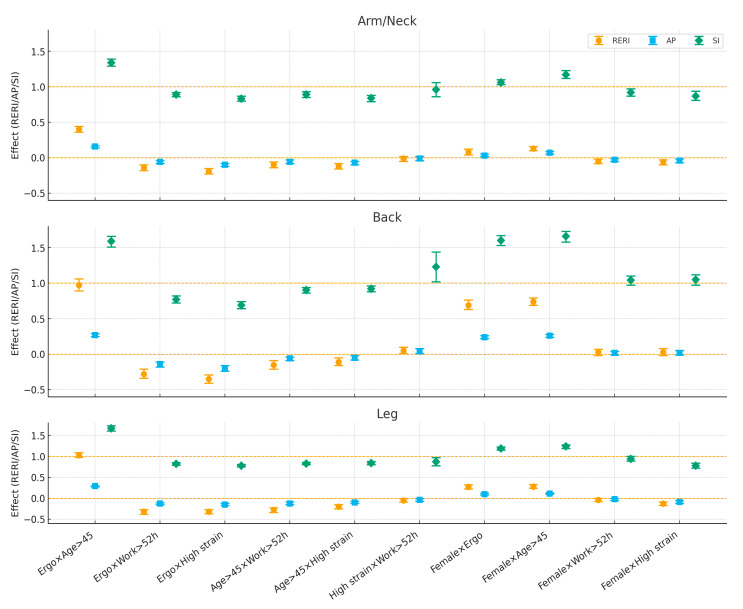
Additive interaction measures (RERI, AP, SI) for pairwise combinations of risk factors by body region in the overall sample. Reference line: 0 (RERI/AP), 1 (SI). Markers show point estimates with 95% CI.

Across all three body regions, *ergonomic risk × age > 45* showed *synergy* on the additive scale: the joint effect exceeded the sum of the separate effects (arm/neck: RERI ≈ 0.40, AP ≈ 0.16, SI ≈ 1.34; back: RERI ≈ 0.97, AP ≈ 0.27, SI ≈ 1.59; legs: RERI ≈ 1.03, AP ≈ 0.29, SI ≈ 1.67; all CIs excluded the null). In contrast, combinations of ergonomic risk *with long working hours* or with *high job strain* displayed *antagonism* (negative RERI/AP and SI < 1), with the largest attenuation for back and leg pain (e.g., ergo × work: RERI ≈ −0.28 to −0.32; ergo × strain: RERI ≈ −0.35 to −0.32). Interactions between *age × long working hours* and *age × high job strain* were modestly antagonistic across regions. The *job strain × long working hours* pair was near-null overall (CIs generally included the null; a small positive departure for back: RERI ≈ 0.05, SI ≈ 1.23) (Figure 2).

**Figure 3:**
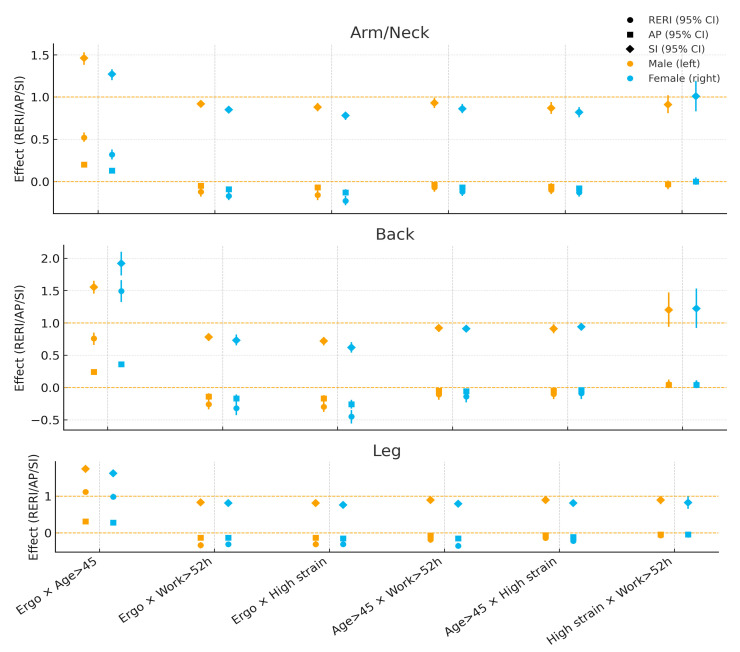
Sex-stratified additive interaction measures (RERI, AP, SI) by body region: Male vs. Female. Reference line: 0 (RERI/AP), 1 (SI). Markers show point estimates with 95% CI.

Patterns 45. showed the strongest *synergy*—for *back pain in women* (RERI ≈ 1.49; AP ≈ 0.36; SI ≈ 1.92) and for *leg pain in men* (RERI ≈ 1.11; AP ≈ 0.31; SI ≈ 1.74); for *arm/neck pain*, synergy was higher in men (RERI ≈ 0.52) than in women (≈0.32). Pairings of ergonomic risk with *long working hours* or *high job strain* were *antagonistic* in both sexes, with attenuation generally *stronger in women*, particularly for arm/neck and back (e.g., ergo × strain for back: women RERI ≈ −0.45, SI ≈ 0.62; men RERI ≈ −0.30, SI ≈ 0.72). *Age × long working hours* and *age × high job strain* also tended toward antagonism in both sexes, again somewhat higher in women (e.g., legs: age × work women RERI ≈ −0.35 vs. men ≈ −0.18). The *job strain × long working hours* pair remained close to null for both sexes (CIs overlapped the null) (Figure 3).

Taken together, older age *amplifies* the impact of high ergonomic exposure on musculoskeletal symptoms (additive synergy), whereas the joint combinations of ergonomic exposure with long work hours or high job strain tend to *dilute* the additional risk on the additive scale—even though each factor independently shows increased odds in multiplicative models. These patterns were consistent across sexes, with notable body-region differences in magnitude.

## 4. Discussion

### 4.1. Prevalence of MSDs in Korea and Comparison with Europe

Our findings revealed that the symptoms of MSDs are highly prevalent, with 35.3% of participants reporting symptoms in the arms and neck, 20.3% in the back, and 24.6% in the legs. More than 40% reported symptoms in two or more regions, underscoring the considerable burden of MSDs in the Korean workforce. Comparison with the European Working Conditions Survey (EWCS) demonstrates that this burden is not unique to Korea. The EWCS reported that 43% of workers experience back pain, 41% report pain in the shoulders, neck, or upper limbs, and 29% report lower limb pain [17]. Both the KWCS and the EWCS have consistently identified MSDs as the most frequent work-related health problem. However, there are meaningful differences: while the prevalence of upper limb and neck symptoms is similar, the prevalence of back pain appears higher in Europe. This may be partly due to differences in survey methods: European surveys rely primarily on self-reported questions, whereas the Korean survey asks about pain only when it is perceived to be work-related. Both the European and Korean surveys conduct face-to-face interviews through home visits, but there are some differences. In Europe, interviewers visit specifically selected individuals, which tends to encourage more active and candid responses. In contrast, in Korea, interviewers visit households and conduct interviews with any employed household member, so other family members may be nearby during the interview, possibly making the respondent feel constrained or less open. Cultural differences between Europe and Korea may also have contributed to these results. In other words, reporting bias may exist—European workers tend to report even minor symptoms, whereas Korean workers may underreport due to a cultural tendency to endure discomfort or concerns about potential disadvantages.

### 4.2. Development of the Korean JEM Compared to International Approaches

In Korea, over 60% of compensated occupational diseases are MSDs, yet regional and inter-expert discrepancies in recognition remain [2]. The absence of standardized benchmarks related to exposure has contributed to these inconsistencies. The development and validation of the Korean JEM represent a significant advance in assessing ergonomic risk. Constructed from self-reported ergonomic exposures by occupational category and validated through expert consensus, the JEM showed high inter-rater agreement (κ ≥ 0.79). Conceptually, this approach parallels JEMs developed in France (CONSTANCES), Finland, and the Netherlands, which also rely on large representative surveys and standardized occupational codes, and undergo expert review [11,12,15]. However, differences exist: the CONSTANCES JEM includes 27 exposures and stratifies by gender, while the Korean JEM employs a simpler classification emphasizing body part-specific risks. European JEMs often incorporate psychosocial and organizational factors, whereas the Korean JEM currently focuses on physical ergonomic exposures. Interestingly, the Korean JEM shows slightly higher agreement between expert ratings and JEM-based classifications for upper body and back exposures compared to some European matrices, supporting its validity [11,12].

### 4.3. High-Risk Occupational Groups in Korea and Europe

As with the KWCS, the EWCS also identified manual labor occupations—such as agriculture, construction, and manufacturing—as high-risk groups for MSDs [17]. In Korea, the high prevalence of musculoskeletal symptoms in multiple sites among crop growers, agricultural/forestry/fishery workers, construction laborers, cleaners, and notably shipbuilding-related workers reflects the country’s industrial structure, wherein shipbuilding remains a significant sector. Conversely, in Europe, where shipbuilding has substantially declined, the healthcare and social service sectors emerge as high-burden occupations [18]. This difference partly stems from variations in industrial composition but also reflects gender-based labor market structures and policies: women’s labor force participation in sectors like healthcare is influenced by factors such as maternity leave and postpartum return-to-work policies. Hence, cross-national variations in MSD prevalence by occupation likely mirror not only differences in work organization and ergonomic standards but also the broader socio-economic and policy contexts that shape workforce composition and gender roles within occupations.

### 4.4. Multivariable Logistic Regression: Independent and Sex-Specific Effects

Multivariable logistic regression confirmed ergonomic risk as the strongest determinant of musculoskeletal symptoms across all body regions, with high-risk workers experiencing more than twice the odds of pain compared with low-risk workers. Age over 45 years was also strongly associated with symptoms, particularly leg pain, while long working hours and high job strain independently increased risks. These associations were stable across sensitivity models, and variance inflation factor diagnostics indicated no problematic collinearity, supporting the robustness of findings. Sex differences were also evident. Women reported a higher crude prevalence of MSDs across all body regions. However, after adjustment, their odds were slightly lower than those of the men. This reversal suggests that the crude female excess is largely attributable to differential distributions of age, working hours, and ergonomic exposures, rather than intrinsic biological vulnerability. Women’s higher crude prevalence appears to reflect their greater representation in jobs and conditions with elevated ergonomic risks.

### 4.5. Synergistic and Antagonistic Interactions

Interaction analyses revealed contrasting patterns between the key occupational risk factors for MSDs. The most consistent and biologically plausible synergy was observed between ergonomic risk and older age (≥45 years); on the additive scale, their combined effect exceeded the sum of individual risks, especially for back pain in women and leg pain in men. This synergy likely reflects the cumulative mechanical wear and tear from prolonged exposure to ergonomic stressors compounded by age-related declines in musculoskeletal resilience, including reductions in muscle strength, joint flexibility, and tissue repair capacity. These findings underscore the critical need for age-tailored ergonomic interventions to mitigate MSD risks among aging workers [18].

By contrast, combinations involving long working hours or high job strain demonstrated antagonistic interactions, wherein joint effects were smaller than expected based on individual contributions. Specifically, attenuation was noted for ergonomic risk combined with long working hours or high job strain, and for age combined with psychosocial exposures. These antagonistic patterns should not be misconstrued as protective; rather, they may result from several complex mechanisms. First, ceiling effects in high-prevalence groups limit the observable increase in risk when multiple exposures coexist [1,19]. Second, overlapping causal pathways—where psychosocial and ergonomic risk factors share biological or behavioral routes influencing development of MSDs—can cause less-than-additive joint effects [20]. Third, selection and adaptation phenomena such as the “healthy worker survivor effect” may operate; workers with greater vulnerability might quit high-demand jobs, leaving a healthier workforce subset experiencing lower combined risks [21]. Further, in demanding work environments, physiological or psychosocial adaptation may moderate symptom reporting or risk manifestation [22]. These findings align with prior research indicating that psychosocial and physical risk factors interact in complex, sometimes non-additive ways, affecting MSD outcomes. Importantly, the stronger antagonistic interactions observed among women suggest gender-specific occupational roles and stress responses require consideration in risk assessment and intervention design. While aging synergistically amplifies ergonomic risk, the combined impact of ergonomic exposures with long working hours or high job strain exhibits attenuation—highlighting the multifactorial and dynamic nature of MSD risk. This calls for integrated prevention strategies addressing ergonomic, organizational, and psychosocial factors tailored by age and sex to effectively reduce the burden of MSD.

### 4.6. Strengths and Limitations

This study has several notable strengths. First, it draws on a large, nationally representative dataset of Korean workers, ensuring generalizability and sufficient power to detect sex-specific and interaction effects. Second, the simultaneous examination of multiple exposures—including ergonomic risk, age, long working hours, and job strain—provides a comprehensive view of MSD determinants and their combined effects. Third, the integration of additive interaction metrics (RERI, AP, SI) with conventional logistic regression enhances interpretability, offering insights into biological interactions beyond multiplicative associations.

Several limitations should also be considered. The cross-sectional design precludes causal inference and limits the ability to disentangle temporal relationships between exposures and MSD outcomes. Reliance on self-reported symptoms and exposures raises the possibility of misclassification, although such misclassification is likely to be nondifferential. Residual confounding may persist despite adjustment, especially with regard to unmeasured psychosocial or lifestyle factors. Furthermore, ergonomic exposures were derived from self-reports rather than direct measurements, which may underestimate heterogeneity within occupational groups. Lastly, although additive interaction metrics are informative, their interpretation remains sensitive to prevalence levels.

### 4.7. Policy and Practical Implications

The findings have important implications for occupational health policy and practice. The strong, independent effect of ergonomic risk underscores the need for targeted ergonomic interventions across industries. The observed synergy between age and ergonomic exposures highlights the particular vulnerability of older workers, especially older women, suggesting that ergonomic programs should prioritize age-sensitive strategies such as task redesign, mechanical aids, and workload adjustments. The predominance of antagonistic interactions indicates that combined exposures do not always amplify risks; rather, complex mechanisms may attenuate effects. This cautions against assuming simple additive burdens and suggests that interventions should address overlapping causal pathways, for example, by integrating ergonomic and psychosocial risk management. At a systems level, the Korean JEM can be further developed and institutionalized as a standardized tool to guide workplace risk assessments and compensation decisions, thereby reducing regional and expert-level inconsistencies. Comparisons with similar global data also highlight the potential of expanding JEM frameworks to incorporate psychosocial dimensions, aligning with European approaches.

### 4.8. Future Research Directions

Future studies should adopt longitudinal study designs to establish causal relationships and clarify the temporal ordering of exposures and outcomes. Objective measures of ergonomic load (e.g., wearable sensors, observational assessments) could complement self-reported exposures and reduce misclassification. Research should also explore psychosocial and organizational factors more deeply, given their potential to interact with physical exposures. Cross-national collaborative studies could help identify cultural or systemic drivers of reporting differences and refine JEM methodologies across contexts. Finally, more detailed sex- and age-stratified analyses are warranted to further elucidate subgroup vulnerabilities and create tailored interventions.

## 5. Conclusions

This study demonstrates that MSDs remain highly prevalent among Korean workers, with patterns broadly comparable to those observed in Europe. Ergonomic exposures, age, long working hours, and job strain are key determinants. However, their joint effects vary: ergonomic risk and age synergistically increase MSD risk, whereas other combinations often show antagonistic or attenuated patterns. Women display higher crude prevalence but lower adjusted odds, reflecting differential exposure distributions rather than intrinsic susceptibility. These findings emphasize the importance of ergonomic interventions, age- and sex-sensitive policies, and standardized exposure assessment tools such as the JEM.

The observed synergy between ergonomic exposure and older age suggests that national prevention programs should prioritize age-tailored ergonomic design, training, and workload management. Furthermore, the validated Korean JEM may serve as a practical framework to enhance workplace risk assessment, compensation consistency, and policy development. Collectively, these insights highlight the need for integrated, evidence-based strategies addressing both physical and psychosocial factors to reduce the musculoskeletal burden in the workforce.

## Figures and Tables

**Table 1:** High-risk occupations with maximum ergonomic exposure (score = 6) ranked by the prevalence of musculoskeletal symptoms in all three body parts.

KSCO		Occupation	Total	Pain in All 3 Body Parts	
1-Digit Code	KSCO Code		n	n	%
6	611	Crop Growers	18,702	6150	32.9
9	991	Agriculture, Forestry, and Fishery Related Elementary Workers	665	211	31.7
6	630	Fishery Related Workers	525	163	31.1
7	782	Construction Related Technical Workers	442	114	25.8
9	941	Cleaners and Sanitation Workers	7469	1691	22.6
6	613	Livestock and Stockbreeding Related Workers	502	100	19.9
7	781	Construction Structure Related Technical Workers	61	12	19.7
6	620	Forestry Related Workers	78	15	19.2
9	910	Construction and Mining Laborers	2082	387	18.6
8	876	Ship Workers and Related Workers	11	2	18.2
7	784	Mining and Civil Engineering Related Technical Workers	84	14	16.7
6	612	Horticultural and Landscape Workers	435	64	14.7
7	741	Die and Mold Makers, Metal Casting Workers, and Forge Hammersmiths	177	25	14.1
9	921	Loading and Lifting Elementary Workers	479	67	14.0
7	783	Construction Finishing Related Technical Workers	2265	301	13.3
9	930	Production Related Elementary Workers	1708	211	12.4
7	772	Broadcasting and Telecommunications Equipment Related Fitters and Repairers	1609	192	11.9
7	743	Welders	976	97	9.9
8	841	Metal Casting and Metal Processing Related Operators	383	38	9.9
7	751	Automobile Mechanics	2004	173	8.6
8	891	Wood and Paper Related Machine Operators	328	28	8.5
8	854	Transportation Vehicle and Machine Related Assemblers	2240	189	8.4
7	792	Plumbers	565	46	8.1
7	730	Wood and Furniture, Musical Instrument, and Signboard Related Trade Occupations	491	38	7.7
7	752	Transport Equipment Mechanics	324	23	7.1
7	762	Electricians	1169	81	6.9
7	742	Pipe and Sheet Metal Makers	44	3	6.8

**Table 2:** Adjusted odds ratios and multiplicative interaction terms for musculoskeletal pain by body region and sex (multivariable logistic regression; 95% CI).

Effect	Both Sexes						Male						Female					
	Arm/Neck		Back		Leg		Arm/Neck		Back		Leg		Arm/Neck		Back		Leg	
	Full OR	95% CI	Full OR	95% CI	Full OR	95% CI	Full OR	95% CI	Full OR	95% CI	Full OR	95% CI	Full OR	95% CI	Full OR	95% CI	Full OR	95% CI
Ergonomic exposure (high vs. low)	2.49	(2.38–2.60)	2.42	(2.30–2.55)	2.51	(2.36–2.66)	1.96	(1.84–2.10)	2.04	(1.89–2.20)	2.00	(1.74–2.30)	2.34	(2.23–2.46)	2.42	(2.26–2.58)	2.33	(2.19–2.48)
Age >45 (vs.<45)	1.57	(1.51–1.63)	1.54	(1.47–1.62)	1.79	(1.71–1.86)	2.03	(1.94–2.13)	2.08	(1.97–2.19)	2.67	(2.55–2.80)	2.12	(2.02–2.22)	2.11	(1.99–2.24)	2.25	(2.14–2.37)
Work >52 h/week (vs. <52)	1.71	(1.63–1.79)	1.72	(1.63–1.83)	1.54	(1.45–1.63)	1.51	(1.43–1.60)	1.48	(1.38–1.59)	1.39	(1.30–1.49)	2.27	(2.16–2.40)	2.24	(2.09–2.40)	2.18	(2.04–2.33)
High job strain (vs. low)	1.55	(1.48–1.63)	1.54	(1.45–1.63)	1.38	(1.31–1.46)	1.36	(1.29–1.45)	1.32	(1.22–1.42)	1.29	(1.21–1.38)	1.76	(1.67–1.87)	1.80	(1.67–1.94)	1.48	(1.39–1.58)
Ergonomic risk × Age >45	1.34	(1.28–1.39)	1.37	(1.29–1.45)	1.30	(1.23–1.39)	1.30	(1.21–1.39)	1.20	(1.11–1.29)	1.69	(1.47–1.96)	1.50	(1.43–1.57)	1.45	(1.36–1.55)	1.54	(1.45–1.65)
Ergonomic risk × Work >52 h/week	0.84	(0.81–0.88)	0.84	(0.79–0.89)	0.84	(0.79–0.89)	0.75	(0.70–0.80)		(0.71–0.82)	0.71	(0.64–0.79)	0.71	(0.67–0.74)	0.69	(0.65–0.74)	0.72	(0.68–0.77)
Ergonomic risk × High job strain	0.79	(0.75–0.83)	0.83	(0.78–0.89)	0.75	(0.70–0.80)	0.71	(0.67–0.76)	0.76	(0.70–0.83)	0.63	(0.57–0.70)	0.74	(0.70–0.78)	0.73	(0.68–0.78)	0.74	(0.69–0.80)
Age >45 × Work >52 h/week	0.90	(0.86–0.94)	0.91	(0.86–0.97)	0.89	(0.84–0.95)	0.82	(0.78–0.86)	0.83	(0.77–0.90)	0.82	(0.76–0.88)	0.72	(0.69–0.76)	0.76	(0.71–0.82)	0.68	(0.63–0.73)
Age >45 × High job strain	0.91	(0.87–0.95)	0.91	(0.85–0.97)	0.91	(0.85–0.97)	0.91	(0.86–0.96)	0.91	(0.84–0.99)	0.92	(0.85–0.99)	0.83	(0.79–0.87)	0.83	(0.77–0.89)	0.83	(0.77–0.89)
High job strain × Work >52 h/week	1.00	(0.95–1.05)	0.95	(0.89–1.01)	1.07	(1.00–1.14)	1.03	(0.97–1.09)	1.04	(0.96–1.13)	1.01	(0.93–1.09)	0.94	(0.89–0.99)	0.90	(0.84–0.97)	0.97	(0.90–1.05)
Female (vs. male)	0.91	(0.87–0.95)	0.91	(0.86–0.95)	0.94	(0.90–0.98)												
Female × Ergonomic risk	0.98	(0.94–1.02)	1.26	(1.19–1.33)	1.03	(0.98–1.07)												
Female × Age >45	1.55	(1.49–1.61)	1.37	(1.31–1.44)	1.55	(1.48–1.63)												
Female × Work >52 h/week	1.13	(1.08–1.17)	1.34	(1.27–1.41)	1.05	(1.01–1.10)												
Female × High job strain	0.88	(0.85–0.93)	0.93	(0.88–0.98)	0.85	(0.81–0.89)												

## Data Availability

The original contributions presented in the study are included in the article. Any further inquiries may be directed to the corresponding author/s.

## Abbreviations

MSDs	Musculoskeletal disorders
JEM	Job Exposure Matrix
CONSTANCES JEM	Cohorte des consultants des Centres d’examens de santé JEM
KWCS	The Korean Working Conditions Survey
OSHRI	The Occupational Safety and Health Research Institute
KSCO	The Korean Standard Classification of Occupations
EWCS	European Working Conditions Survey
VIFs	Variance Inflation Factors
RERI	Relative Excess Risk due to Interaction
AP	Attributable Proportion
SI	Synergy Index
RR	Risk Ratio
